# Theory of Mind Deficits in Individuals at Risk and Early Onset of Bipolar Disorder

**DOI:** 10.1192/j.eurpsy.2025.645

**Published:** 2025-08-26

**Authors:** E. Cesim, M. Demir, M. Eyuboglu, Y. E. Turan, Ö. İlhan, S. Uzman Özbek, B. Yalınçetin, E. Bora

**Affiliations:** 1 Department of Neurosciences; 2 Department of Psychiatry, Dokuz Eylül University, İzmir, Türkiye

## Abstract

**Introduction:**

Impairments in theory of mind, which influence the ability to accurately perceive and comprehend the mental states of oneself and others, play a pivotal role in psychiatric diseases. Understanding these cognitive aspects is crucial for developing targeted interventions and improving overall patient outcomes.

**Objectives:**

The purpose of this study is to examine theory of mind in individuals at risk for bipolar disorder and in the early stages of the disorder.

**Methods:**

Sixty-two individuals with first-episode bipolar disorder (FE-BD) (mean age 21.92±4.58), seventy-eight individuals at ultra-high risk for bipolar disorder (UHR-BD) (mean age 20.5±3.93), and seventy-four healthy controls (HC) (mean age 23.36±5.28) were included in this study. The Hinting Task (HT) and the Reading the Mind in the Eyes Test (RMET) were applied to assess theory of mind.

**Results:**

The groups differed significantly in RMET positive (F(2-200)=5.087, p=0.007), neutral (F(2-200)=4.777, p=0.009) subscores, and total score (F(2-200)=11.267, p=0.000). Similarly, differences were found among the groups in terms of hypomentalization (F(2-174)=5.251, p=0.006), hypermentalization (F(2-174)=4.786, p=0.009), and total scores (F(2-174)=13.292, p=0.000) on the Hinting Task. No significant differences were observed in RMET negative scores among the groups (p>0.05). Both the FE-BD and UHR-BD groups exhibited significantly lower scores than the healthy controls across positive, neutral, and total RMET scores, as well as in total Hinting Task scores (p<0.05). Hypomentalization and hypermentalization errors were higher in the FE-BD and UHR-BD groups than in the HC group (p<0.05).

**Conclusions:**

The results revealed significant differences in theory of mind performance between groups, including RMET scores and Hinting Task results. Individuals with first episode bipolar disorder (FE-BD) and those at ultra high risk for bipolar disorder (UHR-BD) consistently scored lower than healthy controls, emphasizing the need for targeted, early interventions in theory of mind and related cognitive processes. Future studies investigating clinical and neurobiological correlates of theory of mind across different stages of bipolar disorder are needed.

**Disclosure of Interest:**

None Declared

